# Effects of ultrasound-guided nerve hydro-dissection on severe post stroke shoulder pain: a pilot study

**DOI:** 10.3389/fresc.2026.1749973

**Published:** 2026-03-27

**Authors:** Yuge Zhang, Yi Guo, Xiaoli Wu

**Affiliations:** 1 Department of Neurological Rehabilitation, Beijing Bo'ai Hospital, China Rehabilitation Research Center, Beijing, China; 2 School of Rehabilitation, Capital Medical University, Beijing, China; 3 Department of Ultrasound, Beijing Bo'ai Hospital, China Rehabilitation Research Center, Beijing, China

**Keywords:** post stroke shoulder pain, post-stroke neuropathic pain, severe shoulder pain, shoulder rehabilitation, ultrasound-guided nerve hydrodissection

## Abstract

**Background:**

Shoulder pain is a major complication following stroke that substantially compromises patients' quality of life, despite the availability of various therapeutic options.

**Objective:**

This pilot study investigates the efficacy of ultrasound-guided nerve hydrodissection in treating severe post-stroke shoulder pain.

**Methods:**

Twelve eligible subjects were randomly assigned to the nerve hydro-dissection group (*n* = 6) and the control group (*n* = 6). All participants were given standardized shoulder rehabilitation, and the intervention group underwent additional ultrasound-guided brachial plexus hydrodissection. Visual analog scale (VAS), Fugl-Meyer Assessment (FMA) motor subscores of upper extremity and Shoulder Constant-Murley score were assessed at the baseline, and day1, week1, week2, week4 after the treatment.

**Results:**

The VAS scores in both groups had shown a decreasing trend at the four time points and there was a significant difference between the groups (*p* = 0.015, 0.015, 0.004 at the later three time points). The changes in Constant-Murley scores of both groups had shown an increasing trend and there was a significant difference between the groups (*p* = 0.026, 0.002, 0.041 at the later three time points).

**Conclusions:**

Ultrasound-guided nerve hydrodissection shows promising preliminary efficacy for post-stroke shoulder pain in this pilot study, warranting further investigation in larger, sham-controlled trials with longer follow-up.

## Background

Post-stroke shoulder pain (PSSP), affecting 30%–70% of stroke survivors within three months of onset, is a prevalent and debilitating complication that severely compromises patients' quality of life and hinders rehabilitation adherence (1). Its pathophysiology involves multifactorial mechanisms, including central sensitization, peripheral neurogenic injury (e.g., brachial plexus entrapment), and biomechanical abnormalities such as glenohumeral subluxation, which collectively contribute to therapeutic complexity (2, 3). While current interventions—including acupuncture, orthoses, botulinum toxin injections, and neuromodulation therapies (4)—demonstrate partial efficacy in mild-to-moderate cases, approximately 20%–30% of patients with severe PSSP remain refractory to conventional treatments due to unresolved nerve compression and persistent neuroinflammation (5). This unmet clinical need underscores the urgency to explore novel, mechanism-targeted approaches for refractory neuropathic pain in this population.

Emerging as a promising intervention, ultrasound-guided nerve hydro-dissection (US -HD) combines precise neural decompression with therapeutic fluid administration (6, 7). This minimally invasive technique potentially addresses both mechanical nerve entrapment and neuroinflammatory components through targeted perineural injection of normal saline with adjunctive agents (8). Nerve hydro-dissection has recently gained traction as an innovative ultrasound-guided intervention for peripheral neuropathies, employing controlled fluid injection (typically saline with or without adjuvants) to mechanically decompress entrapped nerves while modulating neuroinflammatory microenvironments (8). Despite growing interest in hydro-dissection for peripheral neuropathies, its application in post-stroke neuropathic pain management remains underexplored, particularly regarding long-term efficacy and mechanisms specific to central neurological injury patterns.

Previous studies have identified peripheral neurogenic injury as a potential mechanism in PSSP (9, 10). Evidence suggests that neural compression and perineural fibrosis may perpetuate nociceptive signaling in stroke survivors (9). Although preliminary studies demonstrate the efficacy of peripheral nerve-targeted approaches in reducing neuropathic shoulder pain through dual mechanisms of mechanical nerve liberation and biochemical neuromodulation (11, 12), a critical knowledge gap persists regarding hydro-dissection's therapeutic potential in post-stroke neurological contexts. Notably, no clinical trials to date have systematically investigated this technique's safety profile, optimal injection protocols, or sustained analgesic effects specifically in stroke populations. This study therefore aims to evaluate the hypothesis that ultrasound-guided nerve hydro-dissection can ameliorate PSSP, and to gather preliminary data on safety, feasibility, and potential efficacy signals to inform future, adequately powered trials.

## Methods

### Study design and ethics

This prospective randomized controlled trial (registration ID: MR-11-24-029409, medicalresearch.org.cn) received ethical approval from China Rehabilitation Research Center (CRRC-IEC-RF-SC-005-01).

### Participant selection

Stroke patients with shoulder pain admitted to the Neurorehabilitation Department (September 2021-September 2023), underwent systematic screening. Inclusion criteria comprised: First-ever ischemic/hemorrhagic stroke (1–6 months post-onset) with supratentorial lesions sparing thalamic structures; Shoulder pain onset post-stroke (visual analog scale [VAS] ≥ 6); Age 18–75 years with stable hemodynamic status; Absence of pre-stroke shoulder pathology (6-month pain-free history). Exclusion criteria included: Pre-existing shoulder disorders (adhesive capsulitis, rotator cuff injury, surgical history); Alternative pain etiologies (cervical radiculopathy, fractures, central neuropathic pain); Systemic comorbidities (rheumatoid arthritis, malignancy, renal/hepatic insufficiency), Local contraindications (skin infection, inflammation at injection site).

No *a priori* sample size calculation was performed due to the lack of preliminary data. Patients were randomly assigned to the treatment or control group using a simple randomization method (e.g., a computer-generated random number list). To further protect against bias, the group allocation was prepared and sealed in opaque, sequentially numbered envelopes by a researcher not involved in patient enrollment or assessment. The envelope was opened only after the patient's baseline assessment was completed. All post-intervention assessments were conducted by a trained physician who was blinded to the group assignment. This assessor was not present during the procedure and had no access to the allocation list.

### Intervention protocol

All participants received a standardized, multimodal rehabilitation program delivered once daily, 5 times per week for 4 weeks, consisting of: Physiotherapy (PT): Range of motion exercises (passive and active-assisted), scapular stabilization training, active movement facilitation, and postural control training. Occupational Therapy (OT): Task-oriented training using daily activities (e.g., grasping and moving a cup, rotational trunk reaching). Acupuncture: Administered by a licensed practitioner at local (LI15, TE14, SI9) and distal (LI4, LI11) acupoints. Topical medication: Diclofenac diethylamine emulgel applied to the painful shoulder area twice daily. Each PT, OT, and acupuncture session lasted 30 min, delivered once daily, 5 days per week, for 4 weeks (total of 20 sessions per patient). Exercise intensity was progressed based on individual tolerance under therapist supervision, but remained within the standardized protocol framework. All PT and OT sessions were delivered by licensed rehabilitation therapists with a minimum of 3 years of clinical experience in stroke rehabilitation. Acupuncture was administered by a traditional Chinese medicine practitioner with over 5 years of experience in treating post-stroke complications.

### Ultrasound-guided hydrodissection procedure

The intervention group underwent additional ultrasound-guided brachial plexus hydrodissection using SIEMENS OXANA2 with 12L4 linear transducer (15–18 MHz). Procedural details followed established protocols (6, 13):

Patient Positioning: Supine with cervical roll placement for neutral alignment.

Sonographic Localization: Transverse probe orientation at quadrilateral space level.

Needle Approach: 75 mm Sterile Syringe Needle (in-plane, posterior-to-anterior trajectory).

In hemiplegic patients, prolonged shoulder malpositioning can lead to brachial plexus compression or traction injury (14). The brachial plexus gives rise to the suprascapular, axillary, and subscapular nerves, which provide sensory and motor innervation to the shoulder joint and surrounding structures. Entrapment at the trunk level can therefore generate referred pain to the shoulder region. The supraclavicular approach offers a sonographically accessible window with clear bony landmarks (first rib, scalene muscles) and identifiable vascular structures, allowing for precise, real-time needle guidance. Thus, the primary anatomical target was the peri-neural region of the brachial plexus, specifically the soft tissues surrounding the nerve trunks in the supraclavicular fossa. No intramuscular or intra-ligamentous injections were performed. The intervention was specifically designed to address neural rather than muscular or articular structures, based on the hypothesis that peripheral nerve entrapment is a key contributor to post-stroke shoulder pain (9). Under real-time ultrasound guidance, the needle tip was positioned within the fascial planes immediately adjacent to, but not penetrating, the epineurium of the brachial plexus trunks in the supraclavicular fossa. The goal was to separate the nerve from surrounding fascia using fluid pressure, not needle dissection. By hydrodissecting the peri-neural fascia and adhesions, we aimed to: release mechanical entrapment of the nerve trunks; restore normal gliding between the nerve and surrounding fascial layers; reduce nociceptive input from compressed nervi nervorum.

Injectate: 20 mL of 0.9% normal saline was used for each session. Saline was chosen to isolate the mechanical effect of hydrodissection from potential pharmacological confounders. A dynamic pressure-monitoring technique was employed. An initial 5 mL bolus was injected slowly to confirm extra-neural placement and to observe safe fluid dispersion away from the nerve. The remaining 15 mL was then injected incrementally, with continuous real-time ultrasound monitoring to visualize fascial layer separation and to ensure the injectate surrounded the nerve circumferentially without intraneural spread. Each patient received a single injection session. No repeat injections were administered during the 4-week follow-up period.

Safety considerations: All procedures were performed by a physician with over 10 years of experience in ultrasound-guided interventions. Continuous color Doppler imaging was used to identify and avoid adjacent vascular structures (e.g., transverse cervical artery, suprascapular artery). Patients were monitored for 30 min post-procedure for any immediate adverse events. No complications (e.g., neurovascular injury, local infection, hematoma) were observed.

### Outcome measures and safety

Primary outcomes included: VAS score; Fugl-Meyer Assessment (FMA) motor subscores of upper extremity.

Secondary outcomes encompassed: Shoulder Constant-Murley core. Shoulder functional improvements were operationalized as positive delta values (follow-up minus baseline scores) for Fugl- Meyer and Constant-Murley assessments, with negative deltas indicating pain reduction in VAS.

### Statistical analysis

Continuous variables were expressed as mean ± standard deviation (SD) for normally distributed data or median with interquartile range (IQR) for skewed distributions. In the analysis of the outcomes, within-group comparisons were performed by using the Wilcoxon or Friedman test and between-group comparisons were performed by using the Mann–Whitney *U*-test. Analyses were conducted using SPSS 24.0 (IBM Corp.) and visualized through GraphPad Prism 10.

## Results

**Figure 1:**
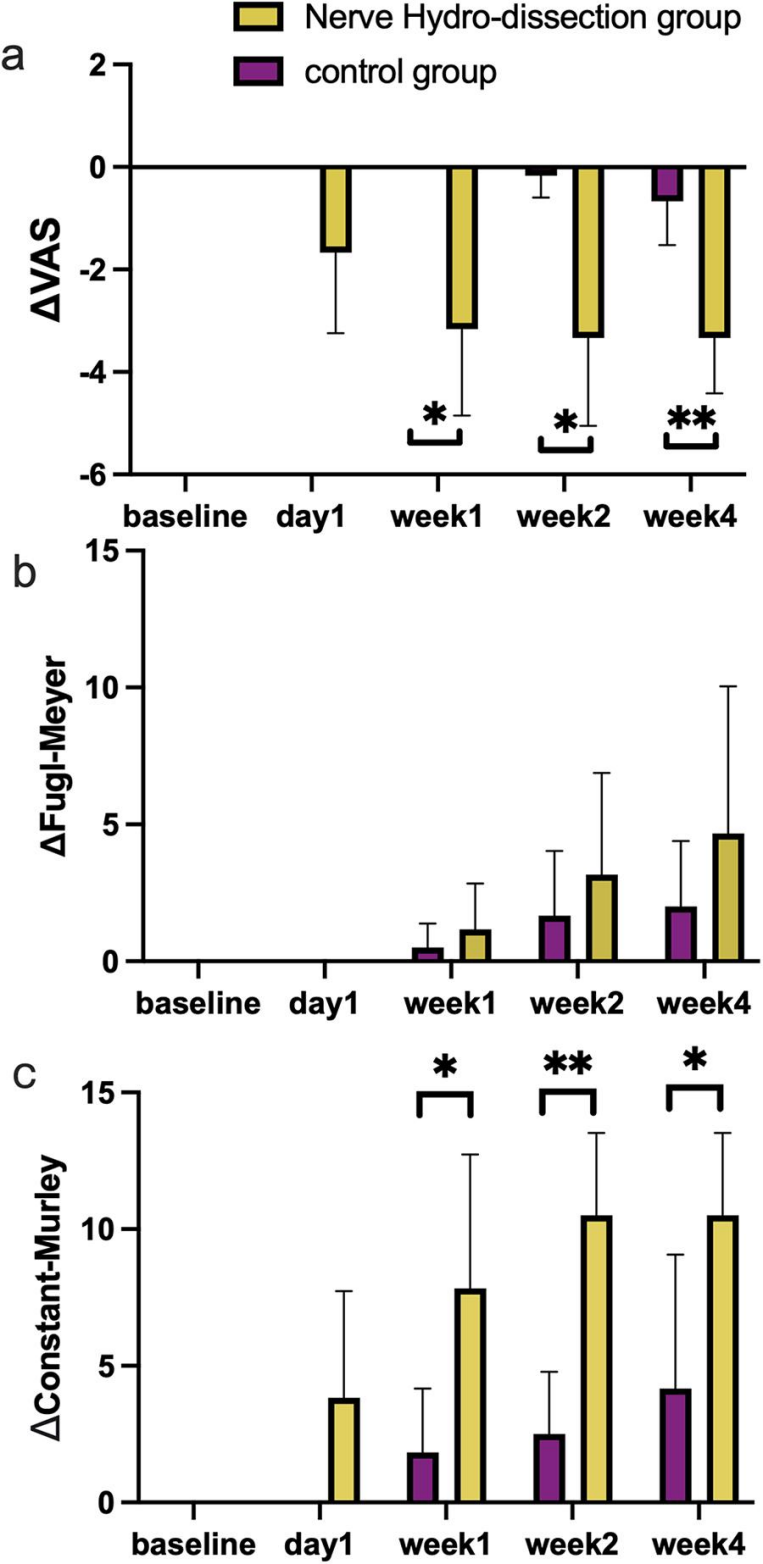
**(a)** Relative changes in visual analogue scale. The baseline pain using VAS was labelled as 0 to normalize the changes in VAS. There is a significant reduction in pain in both groups (*p* = 0.046, 0.002). The pain releases in nerve hydro-dissection group were more than in the control group at week1, week2 and week4 (*P* = 0.015, 0.015, 0.004). **(b)** Relative changes in FMS in the upper limb. The baseline results were labelled as 0 to normalize the changes in the FMS. There was a significant increase in both groups (*p* = 0.004, 0.028), but a non-significant difference among groups (*p* > 0.05). **(c)** Relative changes in CMS. The baseline results were labelled as 0 to normalize the changes in CMS. There is a significant increase in CMS in both groups (*p* = 0.001 for hydro-dissection group, and *p* = 0.007 for the control group). The increases in nerve hydro-dissection group were more significant than in the control group at week1, week2 and week4 (*P* = 0.026, 0.002, 0.041). *p < 0.05, **p < 0.01; VAS, visual analogue scale; FMS, Fugl-Meyer scale; CMS, Constant-Murley scale. Data were presented as mean ± 95% confidence interval (CI). Significance values have been adjusted by the Bonferroni correction for multiple tests.

A total of 97 patients underwent systematic screening, with 12 ultimately enrolled in the trial. Participant Clinical characteristics are summarized in Table 1. All baseline variables showed no statistically significant differences between the two groups (*P* > 0.05 for all comparisons). The main results are illustrated in Figure 1. The VAS scores in both groups had shown a decreasing trend at the four time points (mean ΔVAS = −1.67 ± 0.61, −3.17 ± 0.65, −3.33 ± 0.67, −3.33 ± 0.42 at day1, week1, week2, week4 in the nerve hydro-dissection group, and 0, 0, −0.17 ± 0.17, −0.67 ± 0.33 in the control group) and there was a significant difference between the groups at three time points (*p* = 0.015, 0.015, 0.004 at week1, week2, week4), while the difference at the first time point (*p* = 0.065) did not reach statistical significance. The changes in upper limb Fugl-Meyer scores of both groups had shown an increasing trend (Figure 1B, mean ΔFugl-Meyer = 0, 1.17 ± 1.60, 3.17 ± 3.54, 4.67 ± 5.12 at day1, week1, week2, week4 in the nerve hydro-dissection group, and 0, 0.5 ± 0.84, 1.67 ± 2.25, 2.00 ± 2.28 in the control group) and there was no significant difference between the groups (*p* > 0.5). The changes in Constant-Murley scores of both groups had shown an increasing trend (Figure 1C, mean ΔConstant-Murley = 3.83 ± 3.71, 7.83 ± 4.66, 10.50 ± 2.88, 10.50 ± 2.88 at day1, week1, week2, week4 in the nerve hydro-dissection group, and 0, 1.83 ± 2.23, 2.50 ± 2.17, 4.17 ± 4.66 in the control group) and there was a significant difference between the groups at three time points (*p* = 0.026, 0.002, 0.041 at week1, week2, week4), while the difference at the first time point (*p* = 0.065) did not reach statistical significance.

**Table 1:** Clinical characteristics of participants.

Participant	Participant	Age	Sex	Stroke location	Side affected	Medical history (month)	Initial VAS	Initial Constant-Murley score	Initial Fugl-Meyer score of the Upper limb
Nerve hydro-dissection group	1	55	M	R MCA	Left	3	8	16	9
	2	62	M	L internal capsule	Right	3	8	5	4
	3	51	M	L internal capsule	Left	2	8	19	21
	4	54	M	L MCA	Right	2	8	7	5
	5	54	M	L internal capsule	Right	4	8	11	4
	6	57	M	R MCA	Left	5	10	13	4
Control group	7	68	M	L internal capsule	Right	4	8	5	5
	8	61	M	L pons	Right	3	8	17	18
	9	51	F	L internal capsule	Right	4	8	17	8
	10	60	M	R MCA	Left	2	6	24	11
	11	65	M	R internal capsule	Left	4	10	5	4
	12	65	M	L MCA	Right	2	8	6	5
Statistical value		*t* = −2.128, df = 10				*t* = −0.799, df = 10	*U* = 15.000, *Z* = −0.388	*U* = 15.500, *Z* = −0.407	*U* = 15.000, *Z* = −0.407
*p*		0.059	1.000*	0.545*	1.000*	0.443	0.699	0.699	0.699

* Fisher's exact test; MCA, middle cerebral artery; VAS, visual analog scale.

No neurovascular injuries, local infections, or other procedure-related adverse events were observed among the study participants.

## Discussion

This pilot study provides preliminary evidence that ultrasound-guided nerve hydrodissection (US-HD) may significantly alleviate PSSP and improve functional mobility in the short term, highlighting its potential as a mechanistically targeted approach for peripheral neurogenic pain. These findings align with evidence supporting peripheral nerve decompression in neuropathic conditions [e.g., diabetic neuropathy (15)] and extend prior observations of peripheral nerve injury as a key contributor to PSSP (9). While conventional therapies for PSSP—such as pharmacotherapy, physical modalities, or botulinum toxin injections—may provide symptomatic relief through various mechanisms, they do not specifically target peripheral nerve entrapment. US-HD offers a complementary approach by directly addressing this potential pathophysiological driver, a strategy that, to our knowledge, has not been previously investigated for PSSP. This novelty is critical, as up to 30% of stroke survivors develop refractory shoulder pain resistant to standard therapies, highlighting an urgent need for mechanism-based solutions (5).

### Strategic targeting of the brachial plexus

The selection of the brachial plexus as the intervention site was anatomically and pathophysiologically justified. Prolonged hemiplegic shoulder malposition—characterized by inferior subluxation and adductor spasticity—predisposes the brachial plexus to dynamic compression against the coracoid process and pectoralis minor tendon (14). Moreover, its terminal branches (e.g., axillary and suprascapular nerves) directly innervate the shoulder joint capsule and rotator cuff, transmitting nociceptive signals in PSSP. By hydrodissecting fascial adhesions, US-HD reduces perineural edema and restores vasa nervorum microcirculation, mitigating ischemia-induced axonal dysfunction and toxin accumulation (6). This mechanism parallels findings in carpal tunnel syndrome, where nerve hydrodissection improves intraneural blood flow by >25% on Doppler ultrasound (16).

### Potential mechanisms of action

US-HD exerts therapeutic effects through dual neuromodulatory pathways. Lam et al. (6) comprehensively reviewed the mechanisms and clinical applications of ultrasound-guided nerve hydrodissection, highlighting its ability to alleviate neuropathic pain by decompressing nervi nervorum and vasa nervorum, with evidence supporting 5% dextrose as a preferred injectate due to its dual mechanical and biochemical benefits (6). Lin et al. (11) demonstrated in a retrospective cohort study that ultrasound-guided hydrodissection of cervical nerve roots using a combination of 5% dextrose, lidocaine, and betamethasone provided significant pain relief for cervical radicular pain, with comparable efficacy and safety profiles in patients with mild vs. moderate-to-severe stenosis (11). By injecting fluid to mechanically decompress the brachial plexus from constrictive fascial layers, US-HD not only alleviates mechanical entrapment but also restores vasa nervorum microcirculation, mitigating ischemia and toxin accumulation within the nerve trunk (6). Furthermore, releasing compression on the epineurial “free nerves” may suppress extraneural nociception, thereby interrupting neuropathic pain signaling—a mechanism shared by other nerve-targeted therapies like peripheral nerve stimulation (7, 17). This dual action (mechanical decompression and neuromodulation) aligns with evidence from entrapment neuropathies (e.g., carpal tunnel syndrome), where US-HD achieves sustained analgesia by addressing both structural and functional components of nerve injury.

While the primary rationale for targeting the brachial plexus was to relieve peripheral nerve entrapment, post-stroke shoulder pain (PSSP) involves additional neuromuscular and myofascial factors that may contribute to the therapeutic effects observed. In hemiplegic patients, sustained abnormal postures and muscle imbalance can lead to the formation of active myofascial trigger points (MTrPs) in the rotator cuff and periscapular muscles (18). These MTrPs are characterized by spontaneous electrical activity and serve as independent nociceptive generators that may perpetuate pain even after central neurological injury. Furthermore, MTrPs can exacerbate spasticity through reflex pathways, creating a vicious cycle of pain, increased tone, and mechanical restriction (19). We hypothesize that ultrasound-guided hydrodissection (US-HD) may address these factors through a multilevel mechanism: 1) mechanical decompression of entrapped nerves; 2) disruption of adjacent MTrPs via high-volume fluid pressure, analogous to the local twitch response elicited by trigger point dry needling; and 3) indirect modulation of spasticity through reduced nociceptive input. Thus, US-HD may offer a multifactorial approach targeting both neural and myofascial components of PSSP.

### Postural and biomechanical considerations

Post-stroke shoulder pain is strongly influenced by postural abnormalities and altered scapulothoracic mechanics. In this pilot study, systematic quantitative assessments of posture were not performed, which limits our ability to correlate baseline biomechanical status with treatment outcomes. However, postural correction was an integral component of the standardized rehabilitation program, with therapists providing verbal and tactile cues to promote scapular retraction, neutral spinal alignment, and weight-bearing symmetry (20). Future studies should incorporate objective biomechanical measures and spasticity assessments (e.g., modified Ashworth scale, dynamometry, or electromyography) to better understand these interactions.

### Ultrasound guidance: safety and precision

The ultrasound-guided hydrodissection (US-HD) employed in this study enhances procedural safety by dynamically avoiding neurovascular structures, a critical advantage over blind techniques. In our study, the procedure was well-tolerated with no adverse events reported, confirming the safety of ultrasound-guided injections. Furthermore, ultrasound enabled precise injectate placement between the nerve and constrictive fascial layers, ensuring optimal hydrodissection. This precision is particularly relevant for PSSP patients, who often exhibit altered anatomy due to muscle atrophy or spasticity. The translational potential of US-HD is further supported by its success in other entrapment neuropathies; for example, Fouda et al. (21) conducted a randomized controlled trial comparing ultrasound-guided hydrodissection with open surgery in severe carpal tunnel syndrome, demonstrating comparable clinical efficacy in pain relief and functional improvement while highlighting hydrodissection as a minimally invasive alternative for patients unsuitable for surgery (21).

### Clinical implications

Ultrasound-guided nerve hydrodissection (US-HD) may represent a safe and potentially beneficial option for patients with refractory post-stroke shoulder pain. If confirmed in larger trials, this approach could complement existing rehabilitation strategies to enhance functional recovery and quality of life in stroke survivors.

### Limitations and future directions

The small sample size is a major limitation of this preliminary study, which may limit the generalizability of our findings, and future studies with larger, multicenter cohorts and long-term follow-up are warranted. Given the exploratory aim to assess safety and feasibility, a sham group was not included due to ethical and logistical considerations, but absence of a sham-control group may preclude definitive exclusion of procedural placebo effects. This pilot study relied exclusively on clinical and subjective outcome measures and did not include advanced objective monitoring tools such as ultrasound-based structural evaluation, quantitative spasticity assessment, or kinematic analysis, which limits our ability to correlate clinical improvements with underlying structural or biomechanical changes and to fully characterize the mechanism of action of US-HD. Future studies should incorporate multimodal objective assessment tools—including ultrasound-based structural evaluation, quantitative spasticity measurement, and kinematic analysis—to correlate clinical improvements with underlying structural and biomechanical changes and to elucidate the mechanism of action of US-HD.

## Conclusion

In this preliminary study, US-HD was associated with significant short-term improvement in post-stroke shoulder pain. These findings provide a rationale for future randomized controlled trials to assess the efficacy and durability of this intervention in larger cohorts.

## Data Availability

The raw data supporting the conclusions of this article will be made available by the authors, without undue reservation.
